# Comparative measurement of FeLV load in hemolymphatic tissues of cats with hematologic cytopenias

**DOI:** 10.1186/s12917-019-2208-y

**Published:** 2019-12-19

**Authors:** Mehdi Abdollahi-Pirbazari, Shahram Jamshidi, Seyed Mahdi Nassiri, Mohamad Zamani-Ahmadmahmudi

**Affiliations:** 1 Department of Internal Medicine, Faculty of Veterinary Medicine, University of Tehran, Tehran, Iran; 2 Department of Clinical Pathology, Faculty of Veterinary Medicine, University of Tehran, Qareeb St., Azadi Ave, Tehran, Iran; 3 Department of Clinical Sciences, Faculty of Veterinary Medicine, Shahid Bahonar University of Kerman, Kerman, Iran

**Keywords:** Cat, Cytopenia, Feline leukemia virus, RT-qPCR

## Abstract

**Background:**

Feline leukemia virus (FeLV) is a serious viral infection in cats. FeLV is found in some tissues, such as spleen, lymph nodes and epithelial tissues. However, there is controversy about the organ in which the virus can be reliably detected in infected cats. The purpose of this study was to determine the level of viral infection in hemolymphatic tissues, including blood, bone marrow and spleen by reverse-transcriptase quantitative polymerase chain reaction (RT-qPCR).

**Results:**

A total of 31 cats with clinical signs of FeLV infection associated with at least a single lineage hematologic cytopenia were included in this study. Peripheral blood, bone marrow and spleen samples were obtained from each cat. Complete blood counts, biochemical tests, and a rapid test to detect FeLV p27 antigen in blood samples of cats were performed. Of 31 cats, 9 had anemia alone, 4 had thrombocytopenia alone, 2 had neutropenia alone, 9 had bicytopenia of anemia and thrombocytopenia, 3 had bicytopenia of anemia and neutropenia, and 4 had pancytopenia. FeLV RNA was then detected by RT-qPCR in the whole blood, bone marrow and spleen. Viral RNA copy numbers were detected in all cats by RT-qPCR whereas 24 out of 31 cats were positive for the serum FeLV antigen. We detected a significantly greater number of viral RNA in the spleen compared with the whole blood and bone marrow.

**Conclusion:**

Spleen is a site where FeLV is most frequently detected in cats with hematologic cytopenias.

## Background

Feline leukemia virus (FeLV) is a retrovirus belonging to the family *Retroviridae*, subfamily Orthoretrovirinae, genus *Gammaretroviru*s, which is responsible for infection, tumor development and immunological dysfunction in domestic cats [1–4]. Viremic cats serve as sources of infection for other cats, which can be transmitted via saliva, nasal secretions, urine, feces, and milk [5]. The virus replicates in many epithelial tissues, including salivary glands, oropharynx, esophagus, stomach, intestine, trachea, nasopharynx, renal tubules, bladder, pancreas, alveolar ducts, and sebaceous glands [6, 7]. The virus has two single-RNA strands which are converted to DNA (provirus) by the enzyme reverse transcriptase and integrated into the host cell genome by the integrase enzyme [8]. For this to happen, long terminal repeats in the viral genome play a vital role in the tissue attack and the pathogenicity of the virus [9].

The FeLV is one of the most important pathogens in cats with significant pathologies, which can significantly lower life expectancy in infected cats [10]. During the first few weeks after the initial infection, cats may have the following clinical signs: blood cytopenias (deficiency of any of the various cellular elements normally present in the blood), lethargy and enlarged lymph nodes. Clinical signs depend on viral subgroup and the stage of disease. Common general clinical signs include anemia (pale gums), hyporexia, decreased stamina, depression, diarrhea or constipation, excessive drinking and urination, infertility, jaundice, fever, lymphadenopathy, weight loss, poor coat condition, and neuropathies, with subsequent anisocoria, and hind limb paralysis [11].

Degenerative and neoplastic conditions of the hemopoietic system can be directly attributed to naturally occurring FeLV infection [7]. Hematological disorders, especially cytopenias, due to myelosuppression or myelodysplasia, are common findings in cats infected with FeLV [12, 13], resulting in anemia of myelodysplastic syndrome, aplastic anemia (pancytopenia), transient, persistent and cyclic neutropenias, panleukopenia-like syndrome, and platelet abnormalities [12, 14, 15].

The p27 core viral antigen is the target used for in-clinic diagnostic testing for FeLV, such as rapid immunochromatography and enzyme-linked immunosorbent assays.

In some infection stages, FeLV is found in some tissues, such as cornea, spleen, lymph nodes, or epithelial tissues [7], but not in the bone marrow [8], which is a tissue where infection is usually considered a necessary stage for disease progression. FeLV infection is also associated with viremia, although p27 antigen negativity cannot necessarily rule out the infection [16]. Indeed, more sensitive molecular methods should be adopted when FeLV is suspected [16]. However, there is no comprehensive study on the quantification of FeLV RNA viral load in different tissues of cats with hematological cytopenias. In this study, whole blood, bone marrow and spleen specimens were obtained from 31 cats with hematopoietic cytopenias with suspicion of FeLV infections to detect the presence of the FeLV and to compare the number of viral RNA by an absolute reverse-transcriptase quantitative polymerase chain reaction (RT-qPCR) technique in lymphohematogenic tissues.

## Results

### Characteristics of the study population

Demography of all cases, together with clinical and hematological findings is summarized in Table 1. Serum p27 antigen tests of animals included in this study revealed that 24 out of 31 cats with hematological cytopenias (77.41%) were positive for the presence of FeLV antigen. Meanwhile, by RT-qPCR, viral RNA copy numbers were detected in all the cats (Table 2). Viral RNA could be traced back in all tissues examined, including the whole blood, bone marrow, and spleen. The median age of the FeLV positive feline population in this study was 3.1 year (range: from 1 to10 years). There was a male predominance (21 out of 31, 68%) in this case population. As much as 55% of these FeLV positive cats were sexually intact and 45% were neutered. The breeds of the feline population were domestic short-haired (DSH) and Persian. All cats included in this study were kept indoor, with 6 of them having free access to the outside. Most common abnormal clinical findings in these cats were oral inflammation (55%), fever (55%), and diarrhea (38.7%). None of the patients had simultaneous FIV infection. Nine cats suffered from nonregenerative anemia alone (29.03%), 4 from thrombocytopenia alone (12.90%), 2 from nonregenerative neutropenia (6.45%), 9 from bicytopenia of nonregenerative anemia and thrombocytopenia (12.90%), 3 from bicytopenia of nonregenerative anemia and neutropenia (9.67%), and 4 from pancytopenia (12.90%). Cats of this study with nonregenerative anemia had PCV ranging from 8 to 27% (median, 17.1%), with absolute reticulocyte counts ranging from 1000 to 49,000/μL (median, 11,700/μL). Cats with nonregenerative neutropenia had neutrophil counts ranging from 910 to 1992/μL (median, 1337/μL). Platelet counts in cats with thrombocytopenia ranged from 9000 to 96,000/μL (median, 49,000/μL). Serum biochemistry in cats of our study was normal except for total bilirubin. 20 out of 30 (81%) cats of the study had hyperbilirubinemia ranging from 0.43 to 2.8 mg/dl (reference range: 0.1–0.4 mg/dl) (median: 1.54 mg/dl).

**Table 1:** Demographics of 31 cats included in this study

Cat No	Age (Year)	Gender	Sexual status	Breed	Clinical findings	Hematology findings	Serum FeLV p27
1	2	Male	Neutered	DSH	Oral inflammation, Cachexia, Icterus	Anemia	+
2	2	Male	Intact	DSH	Oral inflammation, Cutaneous lesions, Icterus	Anemia	+
3	5	Male	Intact	Persian	Oral inflammation, Diarrhea, Pale mucous membranes	Anemia, Neutropenia, Thrombocytopenia	+
4	1	Male	Neutered	DSH	Cutaneous lesions, Icterus	Anemia, Neutropenia	+
5	6	Male	Neutered	Persian	Oral inflammation, Purulent nasal discharge, Pale mucous membranes	Anemia	+
6	1	Male	Neutered	Persian	Fever, Cachexia	Thrombocytopenia	+
7	4	Male	Intact	Persian	Oral inflammation, Cachexia, Diarrhea, Pale mucous membranes	Anemia	–
8	10	Female	Neutered	DSH	Oral inflammation, Fever, Icterus	Anemia, Neutropenia	–
9	2	Male	Neutered	DSH	Cutaneous lesions, Diarrhea, Icterus	Anemia	+
10	1	Female	Intact	DSH	Fever, Cachexia, Diarrhea	Anemia, Thrombocytopenia	+
11	3	Female	Intact	DSH	Oral inflammation, Fever, Diarrhea	Thrombocytopenia	+
12	5	Female	Neutered	Persian	Oral inflammation, Fever, Cutaneous lesions, Icterus	Anemia, Neutropenia, Thrombocytopenia	+
13	1	Male	Intact	DSH	Fever, Purulent nasal discharge, Icterus	Anemia, Neutropenia	+
14	4	Male	Intact	DSH	Oral inflammation, Cutaneous lesions, pale mucous membranes	Anemia, Thrombocytopenia	–
15	2	Male	Neutered	DSH	Fever, Icterus	Anemia, Thrombocytopenia	–
16	1	Female	Intact	DSH	Cachexia, Diarrhea	Thrombocytopenia	+
17	2	Male	Intact	DSH	Oral inflammation, Fever, Diarrhea, Icterus	Anemia, Thrombocytopenia	+
18	6	Male	Neutered	Persian	Fever, Cutaneous lesions, Diarrhea	Thrombocytopenia	–
19	4	Male	Neutered	Persian	Purulent nasal discharge, Icterus	Neutropenia	+
20	2.5	Male	Neutered	Persian	Fever, Purulent nasal discharge, Icterus	Anemia, Thrombocytopenia	+
21	1	Male	Intact	DSH	Oral inflammation, Icterus	Anemia	–
22	4	Female	Intact	DSH	Oral inflammation, Fever, Cachexia‚ Diarrhea	Anemia	+
23	3	Male	Intact	DSH	Oral inflammation, Fever, Diarrhea‚ Icterus	Anemia, Thrombocytopenia	+
24	3	Male	Neutered	Persian	Oral inflammation, Icterus	Anemia, Thrombocytopenia	+
25	1	Male	Intact	DSH	Fever, Cutaneous lesions, Diarrhea, Pale mucous membranes	Anemia, Neutropenia, Thrombocytopenia	+
26	2	Female	Intact	DSH	Oral inflammation, Dermatological lesions, Pale mucous membranes	Anemia, Thrombocytopenia	+
27	6	Female	Neutered	Persian	Oral inflammation, Fever, Diarrhea	Anemia	+
28	3	Female	Neutered	Persian	Fever, Purulent nasal discharge, Icterus	Neutropenia	+
29	1	Male	Intact	DSH	Fever, Pale mucous membranes	Anemia	–
30	3	Male	Intact	DSH	Fever, Cutaneous lesions, Pale mucous membranes	Anemia, Thrombocytopenia	+
31	5	Female	Intact	Persian	Oral inflammation, Cachexia, Cutaneous lesions, Diarrhea, Pale mucous membranes	Anemia, Neutropenia, Thrombocytopenia	+

**Table 2:** Number of FeLV RNA (per 20 ng extracted RNA) in the blood, bone marrow, and spleen of cats with hematological cytopenias

Cat No.^1^	Blood	Bone marrow	Spleen
1	10,285	1996	19,422
2	16,710	7329	28,846
3	9864	6633	51,459
4	6308	788	161
5	3069	2242	53
6	13,282	847	9459
7	884	5197	7544
8	450	210	25,581
9	2856	4516	18,869
10	1235	2580	29,669
11	1446	1612	6594
12	1998	6910	11,014
13	1340	5597	28,534
14	287	165	11,179
15	218	113	22,477
16	238	1521	25,991
17	449	2249	1845
18	31	8295	18,023
19	414	1880	9897
20	259	654	10,355
21	85	1036	20,654
22	430	398	8204
23	4000	1840	7928
24	10,443	534	87,661
25	15,835	2827	40,088
26	7103	907	76,304
27	16,584	646	20,421
28	15,354	7732	7062
29	83	69	42,229
30	2163	2603	3078
31	178	2661	32,368

^1^Cat numbers are the same as in Table 1

### FeLV detection in the whole blood, bone marrow and spleen of cats with hematological cytopenias

**Fig. 1:**
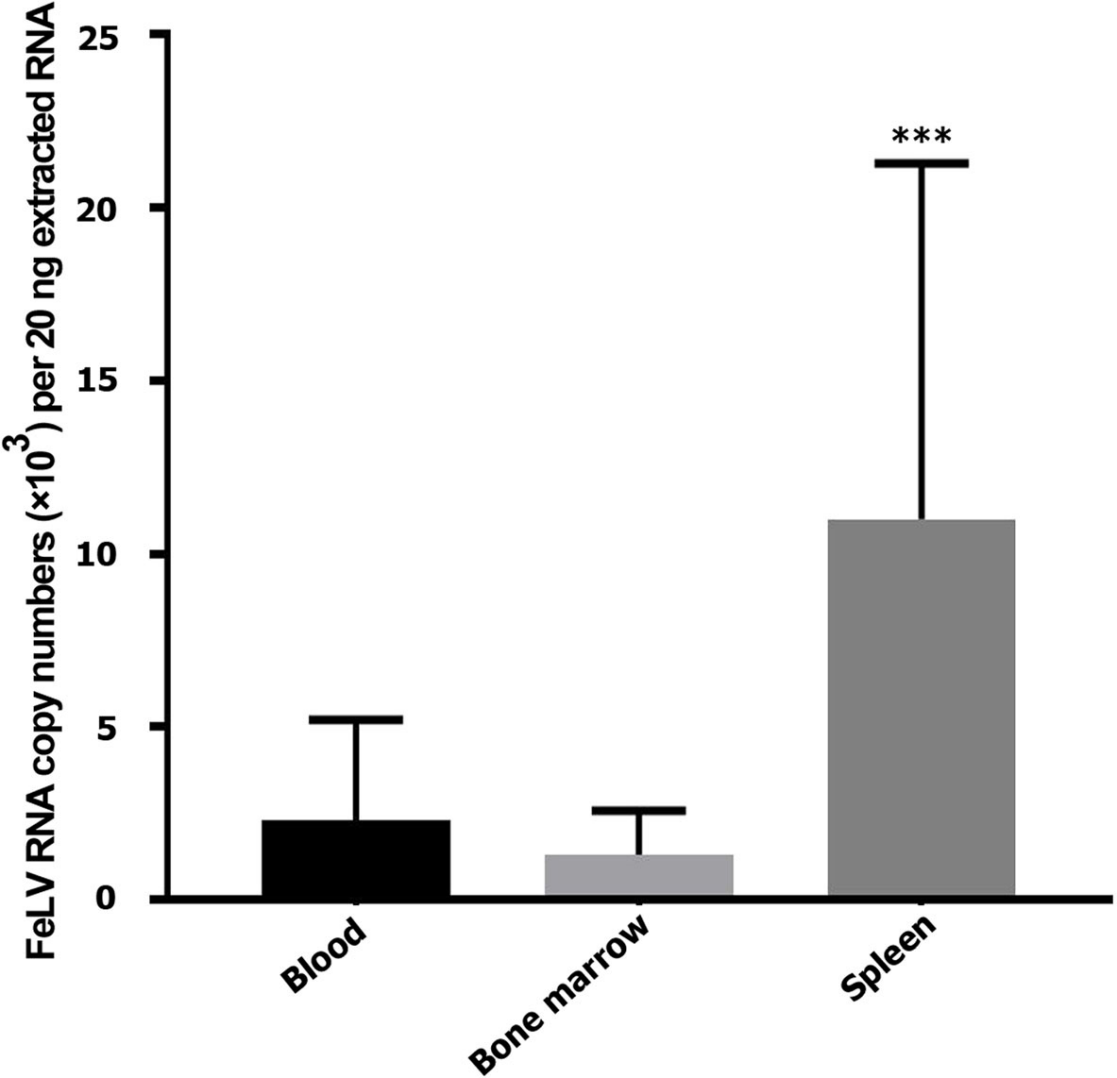
Quantification of FeLV RNA in the blood, bone marrow, and spleen of cats with hematological cytopenias. Data are expressed as mean ± SD. ***P < 0.001, spleen versus blood and bone marrow (One-way ANOVA with Tukey post-hoc)

In this study, we evaluated and compared the number of viral RNA in the whole blood, bone marrow and spleen of cats by an absolute RT-qPCR technique. Our results showed a significantly greater numbers of viral RNA in the spleen (22,031 ± 20,529) compared with the whole blood (4641 ± 5777) and bone marrow (2664 ± 2528) (*P*_(spleen versus whole blood or bone marrow)_ < 0.001) (Fig. 1 and Table 2). We found no correlation between cytopenias and the number of viral RNA in these hemolymphatic tissues.

We determined a cut-off value for viral RNA that resulted in serum p27 antigen positivity. For this purpose, we first ranked patients based on the number of viral RNA in whole blood and then categorized them into four groups (a quartile categorization) [17]. Our analyses revealed that the number of cases with serum p27 positivity in the first quartile was significantly lower than other quartiles (*P* = 0.009), whereas there was no significant differences between other quartiles. Therefore, in quartile 1, a final cut-off value of 94.5 ± 33.4 was determined as the number of viral RNA resulting in p27 antigen positivity in the serum.

## Discussion

The existing literature regarding the quantitative detection of FeLV virus in various reticuloendothelial tissues is profoundly limited. Most of published works about the molecular detection of FeLV have focused on the presence of FeLV in the peripheral blood [1, 2, 4, 18–20]. In this study, we evaluated the presence of FeLV in the whole blood, bone marrow and spleen, and tried to comparatively quantitate the number of viral RNA by RT-qPCR in these lymphohematogenic tissues of cats with hematological cytopenias. Based on circulating proviral DNA and p27 antigenemia, FeLV infected cats were categorized into four classes, designated as abortive, regressive, latent, and progressive [21]. FeLV viremia and viral shedding are considered as evidence of progressive infection, so that molecular detection of provirus, which was carried out by previous studies, might not necessarily lead to recognition of different outcomes of FeLV infection [8]. In the current study, we focused on molecular detection of viral RNA in important hemolymphatic tissues, including bone marrow, infection of which is usually considered a necessary stage for disease progression [8, 22]. The molecular procedure used in this study could successfully detect the virus in the examined tissues of all cats with hematological cytopenias, which is indicative of a progressive infection [23]. Our findings showed that the number of viral RNA in the spleen was remarkably higher than other tissues (*P* < 0.001). We could detect large numbers of FeLV RNA in just 20 ng of total extracted RNA from the spleen, suggesting that even a less invasive fine needle aspiration might have given as effective a reading as a Tru-cut style biopsy for viral detection. It has been shown that the infection first occurs in the oropharynx, where the virus infects lymphocytes, and then the infected cells [24] carry the virus to other target tissues, such as bone marrow, thymus, spleen and lymph nodes [25].

Many authors showed that RNA loads represent ongoing viral replication somewhere in the cat’s body even in sequestered places [8]. Other publications investigated that detection of RNA is generally less frequently accomplished than that of proviral DNA [8].

Due to its availability and quick turnaround time, ELISA is the most common testing method for the detection of FeLV. Previous studies have also shown that only a proportion of cats with FeLV-associated diseases are positive for circulating FeLV antigen [2, 16, 26], which may be due to very low levels of infective virus, transient viremia, or latent or defective forms of FeLV. Indeed, PCR for detection of viral DNA or RNA has been proven to be an ideal procedure for the detection of viral DNA or RNA to diagnose cats with FeLV infection [27].

In this study, we demonstrated the presence of FeLV infection in all the 31 cats with a kind of hematologic cytopenia, including anemia, leukopenia, and/or thrombocytopenia, which is consistent with the association of refractory hematologic cytopenias and myelodysplasia with FeLV infection in cats [2, 28–30]. Indeed, there is some evidence that cats with unexplained peripheral blood cytopenias that have FeLV antigen-negative tests are often suspected to be suffering from latent FeLV infection [13]. In this study, 7 out of 31 cats (22.5%) with a kind of cytopenia were serum p27 antigen negative, indicating further that negativity for FeLV antigen cannot rule out the infection unless the lack of FeLV viral RNA is shown by molecular methods [31, 32].

## Conclusions

Given serum p27 antigen detection has generally lower sensitivity than molecular techniques in FeLV-infected cats, PCR-based molecular methods of viral detection is recommended in suspected cats. According to our findings, spleen should be considered a site where FeLV is most abundantly found in cats with refractory hematologic cytopenias.

## Methods

### Sample collection

A total of 31 cats with clinical signs typical of FeLV-associated immunosuppression, including oral inflammation (gingivitis, stomatitis), fever with unknown origin, chronic purulent nasal discharge without therapeutic improvement, cachexia, cutaneous lesions, chronic diarrhea, and pale mucous membranes were enrolled in this study [33] (Table 1). Cats with at least two of these clinical signs were included in this study as described previously [31]. The cats were subjected to a clinical examination with a Veterinarian at the time of sample collection in addition to filling in a detailed questionnaire. All procedures of this study were approved by the Animal Care Committee of the University of Tehran. Inclusion criteria were as follows: at least one hematologic cytopenia including (1) anemia (PCV ≤30% and absolute count of reticulocytes < 50,000 per microliter), (2) neutropenia (neutrophil counts of less than 2500 per microliter and absolute neutrophil band counts of less than 400 cell per microliter), (3) thrombocytopenia (absolute count of platelets < 100,000 per microliter and no bleeding). Exclusion criteria included FIV positive cats, iron deficiency anemia and thrombocytopenia due to bleeding.

For this study, peripheral blood (3 mL/sample) with or without EDTA anticoagulant, bone marrow and spleen specimens were obtained from each cat. The complete blood counts (CBCs) and biochemical analyses, including total protein, albumin, alanine aminotransferase, aspartate aminotransferase, alkaline phosphatase, total bilirubin, urea, creatinine, glucose, calcium, phosphorus, total cholesterol, and triglyceride were performed using an automated cell counter (Nihon Kohden Celltac alpha, MEK-6450, Tokyo, Japan) and automated chemistry analyzer (Selectra ProM, ELITech Group, Puteaux, France) respectively. A commercially available rapid immunochromatography test (Feline Leukemia Virus Antigen/Feline Immunodeficiency Virus Antibody Test Kit, IDEXX Laboratories, Maine 04092, USA) was performed to detect FeLV p27 antigen in sera of animals.

Bone marrow was obtained from the humerus with a 14-G bone marrow needle under general anesthesia (xylazine 2% (0.5 mg/kg, I.V, Alfesan, Holland) and ketamine 10% (10 mg/kg,I.V Alfesan, Holland) after surgical preparation and disinfection. Bone marrow samples were placed in EDTA tubes.

For biopsy of the spleen, cats were anesthetized as described above. For this purpose, animals were placed in right lateral recumbency, and the area behind the last rib was prepared surgically. We then introduced the biopsy needle (Semi-Automatic biopsy needle, GTA Medical product, Italy) via ultrasound guidance. None of the cats included in this study was subjected to euthanasia after sample collection.

### RNA extraction

Peripheral blood, bone marrow and spleen specimens obtained from each cat were subjected to RNA extraction using a high pure viral RNA purification kit (Cat No: PR891620, AryoGen Biopharma Complex. Karaj, Iran). For this purpose, 100 μl whole blood or bone marrow and 25 mg fresh crushed spleen tissues were added to 1.5 ml micro centrifuge tube containing 400 μl lysis solutions and mixed thoroughly by vortexing for 20 s.

After extraction, RNA concentration was measured at 260 nm with a Nanodrop ND-1000 spectrophotometer (Nanodrop Technologies, Wilmington, DE, USA). Comparable RNA (3.9 ± 0.4 ng/μl) concentrations were determined in the whole blood, bone marrow and spleen samples. After measurement of RNA concentration in each sample, a total of 20 ng RNA was used for RT-qPCR as described below.

### RT-qPCR

FeLV-specific RNA was detected by RT-qPCR (ABI 7700, Applied Biosystems, Foster City, CA) using a commercially available kit (genesig® Advanced Kit. England), which contained FeLV specific primer/probe mix, FeLV positive control template, internal extraction control primer/probe mix, internal extraction control RNA, endogenous control primer/probe mix, FeLV/internal extraction control/endogenous control RT primer mix, RNAse/DNAse free water and template preparation buffer. A FeLV positive control template was provided to generate an absolute standard curve of FeLV copy number (Additional 1: Figure. S1). Also, an RNAse/DNAse free water was used as negative control to validate any positive findings. FeLV specific primer and probe mix provided in the kit was detected using the FAM channel. During PCR amplification, forward and reverse primers were hybridized to the FeLV cDNA. A fluorogenic probe was included in the same reaction mixture which consists of a DNA probe labeled with a 5`-dye and a 3`-quencher. During amplification, the probe is cleaved and the reporter dye and quencher are separated. Amplification was carried out as follows: 55 °C for 10 min (1 cycle) and 95 °C for 2 min (1 cycle), followed by denaturation at 95 °C for 10 s and annealing at 60 °C for 60 s (50 cycles). The standard curve was diluted from 2 × 105 per μl to 2 μl to obtain the quantification range.

### Statistical analysis

Data analysis was performed using SPSS.22 statistical package (Chicago, USA). Frequency of the data was described as mean ± SD values for continuous variables and as proportions for categorical data. Data normality was tested using Kolmogorov-smirnov analysis. One-way ANOVA and Chi-square tests were used to compare groups with continuous variables and categorical data, respectively. The correlation analysis was performed using Spearman’s test. To determine a cut-off value based on the number of viral RNA in blood resulting in serum positivity, patients were first ranked based on the number of viral RNA in blood and then categorized into the four groups (quartile) as described previously for cut-off determination [17]. Following that, the number of cases with the disease between groups was compared using Chi-square test. This analysis revealed that number of cases with FeLV in the quartile 1 was significantly lower than other quartiles (*P* = 0.009). Thus, the mean of blood viral RNA copy numbers in this group was considered as a final cut-off value.

## Supplementary information

**Additional file 1.** Figure. S1.Reverse-transcriptase quantitative polymerase chain reaction (RT-qPCR) of FeLV RNA. (A) 1 × 10^0^ to 1 × 10^− 6^ serial dilutions of FeLV RNA were prepared and detected by real time PCR, with 2 × 10^5^ to 2 × 10^0^ viral RNA copy numbers in the prepared dilutions. (B) Standard curve of the prepared dilutions.

## Data Availability

The data used and/or analysed during the current study are available from the corresponding author on reasonable request.

## Abbreviations

FeLV: Feline leukemia virus
RT-qPCR: Reverse-transcriptase quantitative polymerase chain reaction
