# Supplementary figures and images for: Comparative measurement of FeLV load in hemolymphatic tissues of cats with hematologic cytopenias
Source: BMC Vet Res. 2019 Dec 19;15:460. doi: 10.1186/s12917-019-2208-y (PMC6924046; doi:10.1186/s12917-019-2208-y)

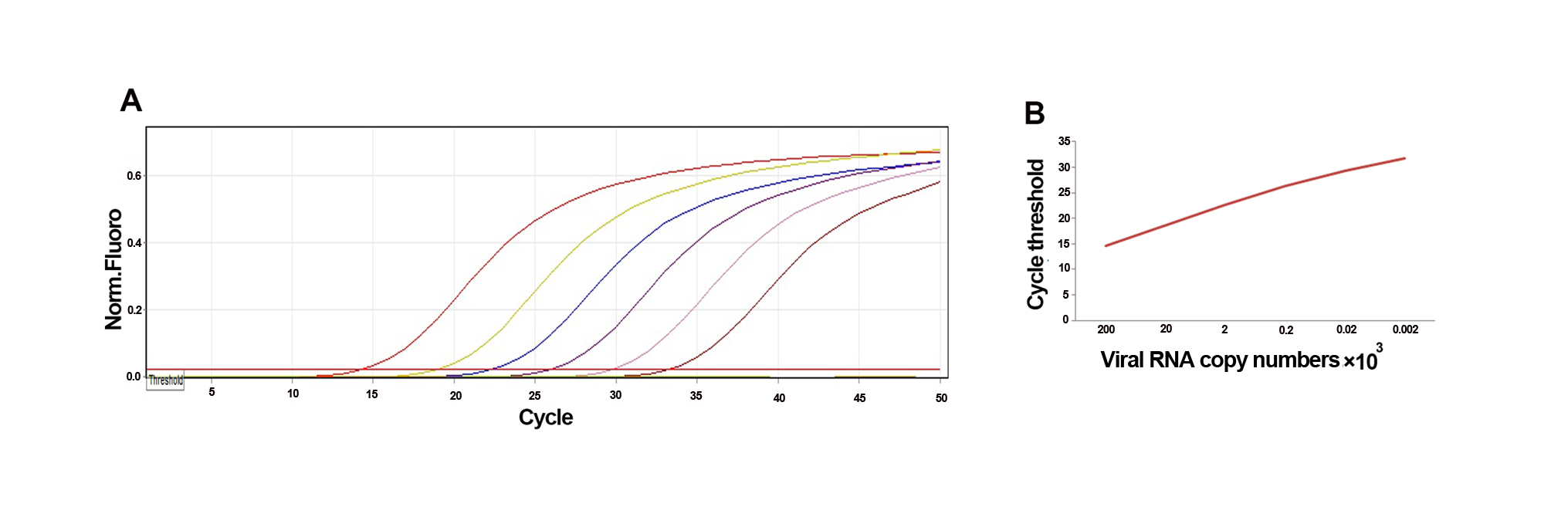

Supplement: Supplementary file 1 — Additional file 1. Figure. S1.Reverse-transcriptase quantitative polymerase chain reaction (RT-qPCR) of FeLV RNA. (A) 1 × 100 to 1 × 10− 6 serial dilutions of FeLV RNA were prepared and detected by real time PCR, with 2 × 10^5^ to 2 × 100 viral RNA copy numbers in the prepared dilutions. (B) Standard curve of the prepared dilutions. [file 12917_2019_2208_MOESM1_ESM.tif]
